# Relationship between Experiential Avoidance and Emotional Disturbances in Coping with Disease in Patients with Multiple Sclerosis

**DOI:** 10.3390/bs14100930

**Published:** 2024-10-10

**Authors:** María Cuerda-Ballester, David Sancho-Cantus, David Martínez-Rubio, Belén Proaño-Olmos, María Pilar García-Pardo, José Enrique de la Rubia Ortí

**Affiliations:** 1 Doctoral Degree School, Catholic University San Vicente Mártir, 46001 Valencia, Spain; maria.cuerda@ucv.es; 2 Department of Nursing, Catholic University San Vicente Mártir, 46001 Valencia, Spain; belen.proano@ucv.es (B.P.-O.); joseenrique.delarubi@ucv.es (J.E.d.l.R.O.); 3 Department of Nursing and Physiotherapy, University of Lleida, 25006 Lleida, Spain; davidmartinezrubio7@gmail.com; 4 Department of Psychology, Universidad Europea de Valencia, 46010 Valencia, Spain; 5 Department of Psychology and Sociology, University of Zaragoza, Campus Teruel, 44003 Teruel, Spain; magarpar@unizar.es

**Keywords:** multiple sclerosis, anxiety, depression, experiential avoidance, psychological inflexibility

## Abstract

Multiple sclerosis (MS) is a neurodegenerative disease that presents with both motor and non-motor symptoms, with anxiety and depression being prominent and potentially exacerbated by negative thoughts. Therefore, the experiential avoidance (EA) exhibited by patients post diagnosis is particularly relevant. This study aimed to measure the degree of EA in patients with MS and determine its relationship with emotional disturbances. A cross-sectional descriptive study was conducted using a sample of 64 patients diagnosed with MS. In October 2018, these patients underwent evaluations of functional and cognitive variables, such as anxiety, depression, and avoidant behaviors towards the disease, using the Expanded Disability Status Scale, Acceptance and Action Questionnaire-II, Self-Compassion Scale Short Form, Five Facet Mindfulness Questionnaire-15, prefrontal symptoms inventory, Beck Depression Inventory II, and State-Trait Anxiety Inventory to assess coping mechanisms in handling the disease. Higher levels of state anxiety (β = 0.79; *p* < 0.001), trait anxiety (β = 0.82; *p* < 0.001), and depression (β = 0.62; *p* < 0.001) were observed in patients with MS as their EA and psychological inflexibility increased. Participants with high self-compassion/self-acceptance tended to have fewer negative thoughts and exhibited better coping with the disease, which may, in turn, affect patterns of psychological rigidity or inflexibility. Dimensions such as kindness and humility could act as positive factors in coping with the disease, whereas self-judgment and isolation are negative elements often associated with avoidant behaviors that hinder effective coping with the illness.

## 1. Introduction

Multiple sclerosis (MS) is a neurodegenerative condition characterized by the formation of demyelinating plaques in the white matter of the central nervous system. These active brain lesions, primarily due to the accumulation of IL-11+ CD4+ cells whose levels are elevated in patients with MS [1], are exacerbated, causing the disease to progress and clinically worsen [2]. Specifically, it presents with both motor and non-motor symptoms, with cognitive impairment and affective disorders such as depression or anxiety being prominent among the latter [3].

Recently, cognitive and psychiatric symptoms in patients with MS have garnered special interest due to the array of deficits caused by the disease and the resulting impact on functional disability [4].

This type of illness directly impacts patients in aspects such as processing the implications of the disease itself or coping with it. Evidence suggests that the negative thoughts commonly expressed by patients following diagnosis are a risk factor for the development of psychiatric disturbances like depression [5,6].

One explanatory theory regarding the emergence and persistence of these negative thoughts is the concept of psychological inflexibility, defined as a rigid thinking pattern [7], which has shown a direct correlation with the severity of symptoms in chronic illnesses (including MS), quality of life, and treatment adherence, indicating a sign of psychological vulnerability. A decline in quality of life and reduced treatment adherence have been observed in such situations [8]. This construct is typically broken down into three main components: experiential avoidance (EA); cognitive fusion (the merging of thoughts, memories, and judgments); and lack of clarity in values (guiding principles of behavior, implying inconsistency or incoherence in conduct).

EA is a symptom frequently observed in patients with MS and can be defined as “the attitude adopted by an individual to avoid exposure to distressing thoughts or images” [9]. According to theories addressing EA, patients are unwilling to experience private events, such as bodily sensations, thoughts, or emotions, and attempt to alter how and where these events manifest [10]. As a consequence, anxiety is temporarily reduced, but effective coping with fears and emotional processing is hindered [11].

This highlights a directly proportional relationship between psychological inflexibility and the presence of anxiety, depression, or stress, underscoring the complexity of managing this symptomatology [12]. EA has been linked to chronic conditions like post-traumatic stress disorder, which often co-occurs with other psychosocial disorders [13].

This avoidance attitude towards the implications of the disease is related to the concept of compassion, specifically self-compassion (also referred to as “self-acceptance”). In its broadest sense, this can be understood as “a feeling of pity and compassion towards those who suffer misfortunes and hardships” [14]. Self-compassionate individuals treat themselves with warmth and empathy in challenging situations [15]. Several studies [14,16] have shown an association between self-compassion and various mental health issues including depression and anxiety.

Different facets of self-compassion have been defined, some positive, such as self-kindness or humility, and others considered negative: isolation, self-directed judgment, and overidentification. These dimensions are related to various aspects (emotional states or moods) of mental health, both positive (happiness or well-being) and negative (stress and emotional problems), and further linked to how the patient attempts to cope with (or avoid) the disease [15,17,18].

The aim of this study was to assess the degree of EA in patients with MS and determine if there is a relationship with emotional disturbances.

## 2. Materials and Methods

### 2.1. Study Design and Population

A descriptive cross-sectional study was conducted to measure experiential avoidance variables at a specific moment. The study participants were patients with MS affiliated with different MS associations in the region of the Valencian Community, Spain.

Inclusion criteria included patients over 18 years old diagnosed with relapsing-remitting MS (characterized by periods of relapses and stability in between), secondary progressive MS (marked by continuous progression with or without occasional relapses) or other variants of the disease such as primary progressive MS for at least a year, treated with glatiramer acetate or beta-interferon, and with no relapse in the past 6 months. This criterion was chosen because these two medications are the most prescribed and virtually the only medications currently used for patients with MS. They have therapeutic functions similar those of immunomodulatory drugs. Including any patient not on this medication could bias the results, as it might have influenced the emotional and psychological states studied in our work.

The exclusion criteria were pregnant or lactating women, patients with dementia, those on antidepressant treatment, and individuals with hormonal disorders affecting the hypothalamic–pituitary–adrenal axis.

Recruitment involved contacting the directors of each center to explain the project and its objectives. This information was then relayed to all patients with MS through the center’s own channels.

A total of 72 patients diagnosed with MS using the McDonald test [19], conducted by neurologists, were subjected to a set of selection criteria.

### 2.2. Instruments

Data were collected from a final sample of 64 patients in October 2018. Questionnaires were administered by nursing staff and neuropsychologists specializing in neurodegenerative diseases, who were part of the research team, and the participants took 20 min to complete. Prior to administering the questionnaires, patients received instructions on how to complete them.

The instruments used for data collection were as follows:

- The Expanded Disability Status Scale (EDSS) was used to assess functional disability in patients with MS [19].
- Acceptance and Action Questionnaire-II (AAQ-II) [20]: A measure of experiential avoidance and psychological inflexibility, assessing aspects such as unwillingness to experience unwanted thoughts and emotions and inability to be present at the moment.
- The Five Facet Mindfulness Questionnaire (FFMQ-15) [21,22]: This evaluates five facets of mindfulness: observing, describing, acting with awareness, non-judging of inner experience, and non-reactivity to inner experience.
- The Self-Compassion Scale Short Form (SCS-SF) [23]: Assesses how often an individual behaves with kindness and affection towards themselves in complex life situations.
- Beck Depression Inventory II (BDI-II) [24,25]: A questionnaire that evaluates the depressive symptoms presented by the patient, primarily of a cognitive nature, although physiological, emotional, or motivational manifestations were also assessed. This version includes symptoms such as agitation, feelings of worthlessness, difficulty concentrating, and loss of energy. Each item of the questionnaire addresses a depressive symptom, and for each item, four alternative statements are provided, ranked from the least to the most severe.
- State-Trait Anxiety Inventory (STAI) [26,27,28]. It measures state anxiety (immediate response to stressful situations) and trait anxiety (relatively stable personality characteristics).
- Prefrontal Symptoms Inventory (PSI) [29]: It is a self-reported questionnaire that assesses cognitive alterations (related to thinking), emotional alterations (affective in nature), and behavioral alterations (conduct-related) in daily activities. This questionnaire is applicable to both the general population and various clinical populations. Scoring is conducted using a Likert scale ranging from 0 (never or almost never) to 3 (always or almost always), whereby scores exceeding 16 indicate an alteration.

### 2.3. Statistical Analysis

Statistical analysis was performed using the SPSS^®^ v.23 software (IBM^®^ Corporation, Armonk, NY, USA, University license). Categorical variables are expressed as proportions. Continuous variables are expressed as mean, standard deviation, median, and interquartile range (IQR). The normal distribution was assessed using the Kolmogorov–Smirnov test. Mean comparisons between groups were conducted using Student’s *t*-test for independent samples or the Mann–Whitney U test for data with a normal or non-normal distribution, respectively.

Linear regression models were constructed to evaluate the interaction of AAQ-II with functional (EDSS) and emotional state (STAI and BDI) data. For each model, anxiety levels, both state (STAI A/S) and trait (STAI A/T), and depression (BDI) were considered outcome variables, while the AAQ-II, along with age, sex, and EDSS, were used as predictor variables. Collinearity was checked using a variance inflation factor less than 2. Independence of errors was assumed with values between 1.5 and 2.5 in the Durbin–Watson test. The level of significance for all analyses was set at α < 0.05.

### 2.4. Ethical Considerations

This study adhered to the principles of the Helsinki Declaration [30] and was approved by the Human Research Committee of the University of Valencia (procedure number H1512345043343). Patients included in the study provided informed consent after being informed of the study’s characteristics. The participants were informed that there would be no harmful effects.

## 3. Results

The study sample consisted of 64 patients treated with glatiramer acetate or beta-interferon, comprising 18 men (28.1%) and 46 women (71.9%). Among these, 44 participants exhibited relapsing-remitting MS (68.8%), 16 demonstrated secondary progressive MS (25%), and 4 had primary progressive MS (6.2%). The age, functional capacity (EDSS), and time elapsed since diagnosis are presented in Table 1.

No statistically significant differences were found between the sexes in any parameter, except mindfulness regarding thoughts, experiences, and actions in daily life (FFMQ-15). This was observed for both the total score and specifically for the facet of describing (naming and describing observed experiences in words without making judgments or conducting a conceptual analysis of them). Women exhibited higher scores in both instances (see Table 2).

Regarding the degree of EA and its relationship with emotional disorders (Table 3), the AAQ-II questionnaire was associated with all emotional state parameters of patients with MS. The models were conducted for each of the emotional parameters as the outcome variable: state of anxiety (STAI A/S), trait of anxiety (STAI A/T), depression (BDI), controlling for sex, age, and disability status (EDSS). In all cases, the coefficients indicated a positive relationship, where higher scores on the AAQ-II questionnaire were associated with greater levels of anxiety (β = 0.79; *p* < 0.001), trait anxiety (β = 0.82; *p* < 0.001), and depression (β = 0.62; *p* < 0.001). For all the models, the explained variability in the outcome variable was approximately 40%. In addition, there was no effect of sex, age, or level of disability (EDSS).

## 4. Discussion

EA is understood as a transdiagnostic construct that can explain disorders such as anxiety or depression and is considered a predictor of these; as such, it cannot be understood outside the context of psychological inflexibility [8,31]. Previous studies have demonstrated a relationship between some components of psychological inflexibility, such as experiential avoidance or cognitive fusion with emotional alterations, such that this type of response is considered maladaptive [32].

On the other hand, clinically, an increase in self-compassion has been associated with a decrease in anxiety and depression levels. The explanation for this finding could lie in Van Tongeren et al.’s evolutionary theory of compassion [33], according to which it would activate a neurobiological system that generates safety and well-being and that opposes self-criticism. The latter factor has been revealed to be a powerful predictor of anxiety and depression, mainly in chronic diseases that generate prolonged stress over time. Sirois et al. [34] proposed that one of the main consequences of self-acceptance is the increase in self-care by patients, something that takes on special relevance when dealing with a neurodegenerative disease such as MS. In addition, evidence points to the fact that subjects with high self-acceptance tend to have fewer negative thoughts and present better coping with the disease, which, in turn, influences patterns of rigidity or psychological inflexibility [16,35].

In our study, we verified that there is a direct relationship between avoidant attitude and self-acceptance and that there are significant differences with respect to the different dimensions of the “self-acceptance” construct. However, these differences did not appear to be significant in the dimensions of humility in the male sample. A justification for this finding could be the reduced sample size with respect to men [35] and issues related to coping with this type of disease in Latin culture, although there are no studies that have validated these hypotheses in patients with MS.

The different components of self-acceptance evaluated using the SCS-SF showed results that were consistent with what could be expected. The two positive dimensions of the construct (self-kindness and humility) correlated inversely with experiential avoidance, since lower scores on these variables implied a greater presence of avoidant behavior, mainly in the women’s group and in the total scores. With respect to the negative dimensions (self-judgment and isolation), a direct correlation was observed, since higher scores implied more avoidance. In addition, consistent with what appears in the literature, it is usually women who present with higher levels of EA, which gives them greater psychological rigidity that will impact coping and disease evolution [25,35].

Even though the previous literature describes an alteration in executive functions [36], composed of inhibition, flexibility, and working memory factors in patients with MS, this finding has not been reflected in our results. However, studies exploring cognitive alterations in patients with MS are limited. An attempt has been made to correlate these functions with other clinical variables such as depression, although no significant differences have been found [4].

The differences observed regarding sex may be explained by the small sample size of the male group, as there was statistical significance in the total scores that corroborates the described correlation.

It is worth considering whether the treatment with interferon or glatiramer acetate (the most commonly prescribed medications for the disease) received by the patients included in the study could influence their emotional state. In this regard, it is the interferon treatment that generates some controversy due to its complex mechanism of action [37], and it is not clear to what extent the risk of experiencing emotional deterioration is due to the drug and to what extent it is due to the clinical condition for which it was prescribed. However, there is considerable consensus on the fact that this treatment for MS patients is not significantly associated with higher rates of depression [38,39,40], even when there is an increase in patient disability due to disease progression [41].

Based on the findings obtained in the present study, the development and implementation of psychological interventions aimed at improving variables such as psychological flexibility are proposed; using non-pharmacological therapies, an example of this could be Mindfulness Acceptance-Based Interventions that improve and work with variables such as acceptance, psychological flexibility, meditation or mind-fulness [42]. In patients with MS, these interventions based on mindfulness and acceptance practice could improve quality of life, self-management/lifestyle, psychological flexibility, distress reduction, adaptation enhancement, and treatment adherence, as well as coping with the illness [43,44]. This would involve a broader coping process, for example, on one hand, the acceptance of the disease itself and, on the other, the management of emotions and self-regulation, thereby facilitating a focus on the present moment [45].

Concerning the relationship with MBIs and their relationship with experiential avoidance or cognitive changes, there is evidence of there being a potential mechanism of change in MBIs [46,47]. Research has shown that MBI training enhances cognitive abilities (e.g., cognitive flexibility, attention, and executive functioning), which might affect social functioning and quality of life [48,49,50]. In this sense, this type of training or programs could substantially benefit MS patients in different conditions.

As regards the relationship between mindfulness and self-compassion, they are not only theoretically but also empirically related constructs, and both skills are relevant aspects of CBIs and MBIs. In fact, mindfulness is considered a core aspect of self-compassion [51,52,53,54]. In this same sense, although MBIs do not teach specific content and compassion-based meditations, this construct is taught implicitly as an attitudinal foundation of mindfulness practice, and it is mainly conveyed through the instructor’s way of relating to the participants [55]. Furthermore, it has been shown that this type of intervention enhances its effects on this variable [56] and that it can act as a potent mediator of MBIs on depressive symptoms and rumination [57]. Interestingly, the potential mediating role of self-compassion observed might extend the rather modest evidence of this variable as a mechanism of change [58,59].

These results could suggest that explicitly teaching self-compassion skills might increase the effect of MBIs by incorporating this potential mechanism in the therapeutic process with more strength [57].

### Limitations

The main limitation of this study was the sample size regarding the male group, partly due to the fact that MS is more prevalent in women. In addition, the way the data were collected intentionally could constitute another weakness of this study.

On the other hand, it would be interesting to compare our results with those that could be collected from a sample of patients being treated with other drugs such as natalizumab or ocrelizumab, which are currently being administered to MS patients and are effective in modifying the disease at a symptomatic level as they are capable of producing a modulating effect on inflammation. Along the same lines, it would also be interesting to have a control group of healthy individuals to help draw more conclusions.

Mood disorders (anxiety and depression) were diagnosed based on subjective questionnaires and not through a comprehensive psychiatric evaluation.

## 5. Conclusions

In this study, the presence of EA in patients with MS worsened patients’ ability to cope with the disease. The presence of emotional symptoms such as anxiety or depression has been shown to be greater in patients who experience avoidance. Dimensions such as kindness or humility could act as positive factors in coping with the disease, whereas self-judgment or isolation would be negative elements that usually occur along with avoidance behaviors that hinder effective coping with the disease.

These findings highlight the relevance of incorporating psychological interventions into medical or pharmacological treatments that affect variables such as psychological flexibility, compassion, or emotional regulation, as complementary coping strategies to the usual ones and that contribute to improving the quality of life of these individuals.

In this sense, it could be useful to implement Mindfulness Acceptance-Based Interventions, which would also include elements of compassion or compassion programs adapted to this population.

## Figures and Tables

**Table 1:** Description of initial characteristics of the sample of patients with MS.

	M	SD	Me	IR
Age (years)	46.8	11.9	47.0	15.8
edss (functional disability)	3.5	2.1	3.5	4.0
Time since diagnosis (months)	13	9.6	12	14

SD: standard deviation; edss: Expanded Disability Status Scale; M: mean; Me: median; IR: interquartile range; MS: multiple sclerosis.

**Table 2:** Analysis of the main variables by sex in patients with MS.

	Men (n = 18)				Women (n = 46)				*p*-Value
	M	SD	Me	IR	M	SD	Me	IR	
**aaq-ii**	23.2	10.4	21.0	14.5	24.3	10.3	23.5	16.0	0.610
scs_sf_Total	36.1	10.1	35.0	14.5	37.7	5.2	38.5	6.0	0.183
scs_sf_Kindness	6.2	2.2	6.5	3.3	6.7	1.7	6.0	2.0	0.941
scs_sf_Judgment	5.5	3.0	4.5	5.5	5.9	2.3	6.0	3.3	0.268
scs_sf_Common humanity	6.2	2.2	6.0	2.3	6.2	2.2	6.0	4.0	0.957
scs_sf_Isolation	5.7	2.5	5.0	3.0	6.0	2.1	6.0	2.3	0.108
scs_sf_Mindfulness	6.7	2.5	6.5	4.5	6.7	2.1	7.0	2.0	0.878
scs_sf_Over-identification	5.8	2.3	5.0	4.0	6.3	2.4	6.0	4.3	0.393
ffmq-15_Total	40.8	7.9	42.0	10.0	45.9	6.9	46.5	7.5	0.025
ffmq-15_Observing	8.7	3.1	9.0	4.5	9.4	2.9	10.0	5.0	0.324
ffmq-15_Describing	8.7	1.8	8.5	3.0	9.8	1.5	10.0	2.0	0.023
ffmq-15_Awareness	7.7	2.9	7.5	5.0	9.3	2.7	9.5	4.0	0.084
ffmq-15_Nonjudging of experience	7.1	2.7	7.0	4.3	7.6	3.1	7.0	5.0	0.506
ffmq-15_Nonreactivity to inner experience	8.7	3.3	8.5	4.3	9.8	2.9	10.0	4.0	0.320

aaq-II: Acceptance and Action Questionnaire-II; SD: Standard deviation; M: mean; Me: median; ffmq-15: Five Facet Mindfulness Questionnaire; IR: interquartile range; scs-sf: Self-Compassion Scale Short Form; MS: multiple sclerosis.

**Table 3:** Regression coefficients for the prediction of anxiety and depression based on AAQ-II and SCS-SF in patients with MS.

**Variable**	**stai a/s**					**stai a/t**					**bdi**				
**Coefficients**	**b**	**SE**	**β**	***t***	***p***	**b**	**SE**	**β**	***t***	***p***	**b**	**SE**	**β**	***t***	***p***
aaq-ii	0.79	0.13	0.62	6.09	<0.001	0.82	0.12	0.66	6.79	<0.001	0.62	0.10	0.64	6.43	<0.001
Sex	−2.93	3.00	−0.10	−0.98	0.333	0.40	2.78	0.01	0.14	0.886	1.39	2.20	0.06	0.63	0.532
Age	−0.12	0.12	−0.11	−1.05	0.298	−0.02	0.11	−0.02	−0.19	0.849	0.01	0.09	0.01	0.06	0.956
edss	0.33	0.68	0.05	0.49	0.625	0.50	0.63	0.08	0.80	0.425	0.25	0.50	0.05	0.51	0.615
Model	R^2^ = 0.406; F _4, 59_ = 10.1; *p* < 0.001					R^2^ = 0.461; F _4, 58_ = 12,6; *p* < 0.001					R^2^ = 0.433; F _4, 59_ = 11.25; *p* < 0.001				
**Variable**	**stai a/s**					**stai a/t**					**bdi**				
**Coefficients**	**b**	**SE**	**β**	***t***	***p***	**b**	**SE**	**β**	***t***	***p***	**b**	**SE**	**β**	***t***	***p***
scs-sf (Total)	0.30	0.27	0.14	1.08	0.284	0.44	0.26	0.22	1.70	0.095	0.42	0.20	0.27	2.12	0.038
Sex	−2.19	3.96	−0.07	−0.55	0.583	0.17	3.76	0.01	0.05	0.964	0.87	2.87	0.04	0.30	0.764
Age	−0.13	0.15	−0.11	−0.83	0.408	0.00	0.14	0.00	0.02	0.987	0.03	0.11	0.03	0.23	0.817
edss	1.05	0.85	0.17	1.23	0.224	1.19	0.81	0.20	1.47	0.148	0.76	0.62	0.17	1.23	0.223
Model	R^2^ = 0.05; F _4, 58_ = 0.79; *p* = 0.535					R^2^ = 0.082; F _4, 58_ = 1304; *p* = 0.279					R^2^ = 0.10; F _4, 59_ = 1.64; *p* = 0.177				

aaq-ii: Acceptance and Action Questionnaire-II; b: regression coefficient; β: standardized b coefficient; bdi: Beck Depression Inventory; edss: Expanded Disability Status Scale; SE: standard error; F _i, j_: F statistic and its degrees of freedom i, j; *p*: *p*-value; R^2^: determination coefficient; scs-sf: The Self-Compassion Scale Short Form; stai: State-Trait Anxiety Inventory.

## Data Availability

Data can be requested from the corresponding author.
